# Subperineural ‘onion-peeling’ dissection for maximizing extent of resection and facial nerve preservation for onco-functional balance in vestibular schwannoma surgery

**DOI:** 10.1007/s11060-026-05755-5

**Published:** 2026-08-25

**Authors:** James K. Liu, Ahmed Sabra, Yaxel Levin-Carrion, Caryn J. Ha, Sraavya Anne, Arevik Abramyan, Vicente Rojas-Navas, Kevin Wong, Yu-Lan Mary Ying, Robert W. Jyung

**Affiliations:** 1 Department of Neurosurgery, Cooperman Barnabas Medical Center, RWJ Barnabas Health, Livingston, NJ USA; 2 Skull Base Institute of New Jersey, Neurosurgeons of New Jersey, Livingston, NJ USA; 3 Department of Neurological Surgery, Rutgers New Jersey Medical School, Newark, NJ USA; 4 Department of Neurosurgery, School of Medicine, Pontificia Universidad Católica de Chile, Santiago, Chile; 5 Department of Otolaryngology-Head and Neck Surgery, Rutgers New Jersey Medical School, Newark, NJ USA; 6 Department of Neurosurgery, Skull Base Institute of New Jersey Neurosurgeons of New Jersey, Cooperman Barnabas Medical Center, 200 S Orange Ave, Ste 265, Livingston, NJ 07039 USA

**Keywords:** Vestibular schwannoma, Acoustic neuroma, Subperineural, Subperineurial, Facial nerve preservation, Onco-functional balance, Skull base surgery

## Abstract

**Introduction:**

Planned subtotal resection (STR) of vestibular schwannomas (VS) followed by stereotactic radiosurgery (SRS) has gained popularity for minimizing facial nerve injury. However, recurrence risk correlates with residual tumor volume, and long-term outcomes remain unknown. The authors report their facial nerve outcomes with planned maximal safe resection using a facial nerve-sparing subperineural “onion-peeling” technique, and compare their outcomes to those of the hybrid STR-SRS approach.

**Methods:**

A retrospective study was performed on 146 patients who underwent VS resection by a single surgeon using a subperineural “onion-peeling” technique, leaving a thin layer of perineurium as an anatomic buffer overlying the facial nerve. Tumor size, operative approach, extent of resection (EOR: GTR 100%, NTR 95–99%, STR < 95%), facial nerve outcomes by House-Brackmann (HB) grade, recovery time, recurrence, and postoperative SRS usage were analyzed.

**Results:**

Most tumors were Koos grade III-IV (69.9%), followed by grade I-II (30.2%), removed via retrosigmoid (86.3%) or translabyrinthine (13.7%) approaches. *At last follow-up (mean follow-up = 89.8 months)*,* 95.2% of patients achieved HB I-II scores while obtaining an EOR ≥ 95% (GTR/NTR) in 97.3% of patients.* There was no significant difference in rates of HB I-II outcomes whether EOR was GTR or NTR (97.8% vs. 92.5%, *p* = 0.196). HB I-II outcomes were achieved in 100% of Koos I-II and 93.1% of Koos III-IV tumors. There was a trend of decreased GTR and increased NTR with increasing Koos grade I to IV (GTR: 93.75%, 86.2%, 76.9%, 39.5%; NTR: 6.25%, 13.8%, 23.1%, 55.3%, respectively). Postoperative CSF leak rate was 2.7%. Tumor recurrence (3.4%) was lower with GTR than NTR (1.1% vs. 7.5%, *p* = 0.0646).

**Conclusion:**

The subperineural “onion-peeling” dissection technique provides a safe, nerve-sparing approach for maximal VS resection while preserving facial nerve function thus achieving excellent onco-functional outcomes. Compared to historical hybrid STR-SRS data, our approach demonstrated favorable facial nerve outcomes with higher EOR, lower tumor recurrence, and less radiation usage.

## Introduction

Vestibular schwannomas (VSs) are benign Schwann cell-derived tumors that typically arise from the vestibular nerves. They account for approximately 6–8% of all intracranial tumors and are the most common tumor discovered in the cerebellopontine angle (CPA) [1]. Due to the close proximity of these tumors to the facial nerve, the primary goals of VS surgery are to achieve maximal safe tumor removal while preserving facial nerve function, and optimizing long-term onco-functional outcomes and quality of life. However, several studies have suggested that gross total resection (GTR) in patients with large VS is associated with a higher risk of neurological morbidity, particularly with respect to facial nerve injury [2].

Recently, the adaptive hybrid approach, characterized by planned subtotal resection (STR) followed by stereotactic radiosurgery (SRS) has gained popularity as a method to preserve facial nerve function while minimizing the risks associated with aggressive gross total resection (GTR) [2, 3]. While this approach appears favorable in terms of achieving good facial nerve outcomes, several disadvantages include the underestimation of residual tumor left behind and that all patients receive additional radiation therapy. Moreover, the hybrid approach lowers the technical demand and risks diminishing advanced microsurgical expertise in facial nerve dissection for CPA surgery, particularly in non-specialized centers [4]. Furthermore, an exclusively intracapsular debulking approach not only complicates the precise intraoperative assessment of tumor volume reduction but also can increase the risk of residual tumor regrowth and recurrence due to a wide spectrum of STR tumor volumes [5–8]. Notably, a study of 400 patients found that the STR group had a significantly higher rate of tumor regrowth (22%) compared to the GTR group (3%) [5]. While preliminary studies on this nerve-centered strategy report favorable short-term facial nerve outcomes, these approaches lack long-term data on tumor recurrence and fail to address the variability and wide spectrum of STR volumes [9–11].

Rather than adopting pre-planned intracapsular STR, our philosophy has been to perform VS surgery with the intent of GTR using a facial nerve-sparing subperineural (or subperineurial) “onion-peeling” technique. The decision to leave residual tumor is based on intraoperative observation and nuanced clinical judgement. When a decision is made to leave residual tumor behind, the goal is to leave the least amount of residue possible, so that in essence, one can achieve a *radical NTR*. In this study, we report our facial nerve outcomes and extent of resection (EOR) using the subperineural “onion-peeling” technique. This technique uses the perineural membrane (PM) of the vestibular nerves as a dissection plane to allow safer separation of the tumor from the facial nerve while acting as a buffer and shield to protect the vascularity and integrity of the facial nerve. *We hypothesize that this technique can achieve both improved facial nerve outcomes while maximizing volumetric tumor resection thus resulting in optimal onco-functional balance.* The main objective of this investigation was to evaluate facial nerve outcomes with respect to extent of resection using the subperineural technique. While hearing preservation is an important goal in contemporary VS surgery in patients with serviceable preoperative hearing (AAO-HNS Class A or B), this study focused specifically on facial nerve outcomes and extent of resection. The vast majority of patients in this study were larger Koos grade III and IV tumors with non-serviceable hearing (Class C or D) preoperatively, making facial nerve preservation the primary functional goal. Therefore, our definition of onco-functional outcomes primarily refers to postoperative facial nerve function.

## Methods

### Patient selection

A retrospective cohort analysis was conducted to evaluate patients who underwent VS resection using the subperineural dissection technique by a single surgeon between September 2009 to February 2025. Patients with Neurofibromatosis Type II (NF2) were excluded from the final analysis.

### Ethical approval

This study was conducted in accordance with the Declaration of Helsinki. This study was reviewed by the Institutional Review Board (IRB) at Rutgers University, which granted an exemption/waived the requirement for informed consent because the research involved only de-identified, secondary data (Protocol # Pro2013002841).

### Data collection

Data were extracted from electronic medical records and de-identified for analysis. Primary data points included patient demographics, tumor characteristics (Koos Grade, tumor volume), operative approach, EOR, intraoperative facial nerve monitoring, House-Brackmann (HB) scores pre- and post-operatively, facial nerve function recovery time, length of follow-up, complications, and rates of recurrence [12, 13]. HB Grades I to II were considered favorable facial nerve outcomes, while grades III to VI were regarded as unfavorable outcomes. Tumor recurrence was identified as radiographic evidence of regrowth or progression detected on follow-up imaging. Cases of recurrence were managed with SRS or surgical reoperation.

### Subperineural “onion-peeling” dissection technique

**Fig. 1:**
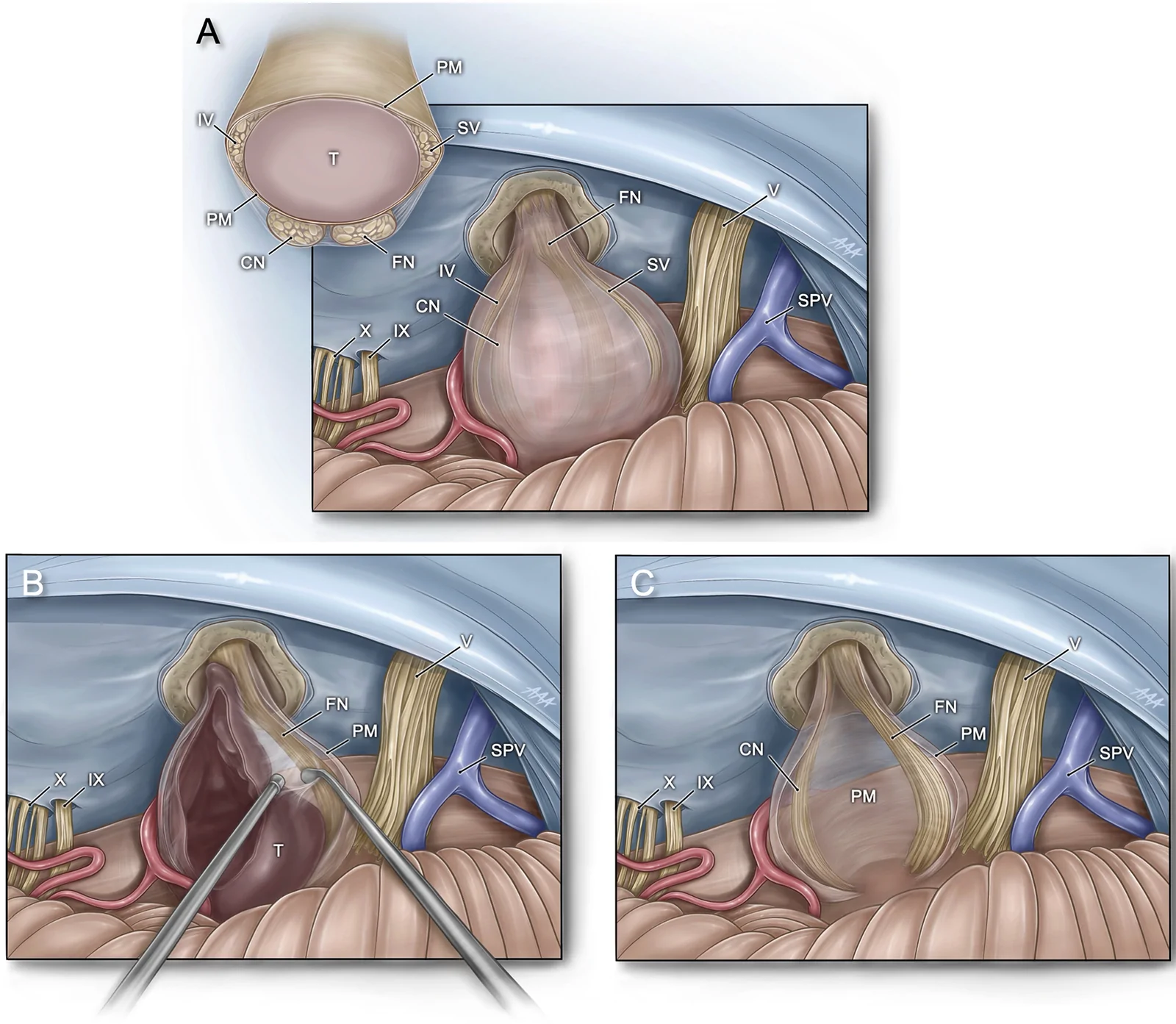
(**A**-**C**): Illustration demonstrating the subperineural “onion-peeling” dissection technique. **A**: The tumor (T) is ensheathed by the perineural membrane (PM) which is an attenuated layer from the perineurium of the superior vestibular (SV) and inferior vestibular (IV) nerves. **B**: After central debulking of the tumor, the tumor is carefully peeled away from the PM which acts as a buffer layer of protection from the facial nerve (FN) and cochlear nerve (CN) that resides on the ventral side of the PM. **C**: After tumor removal, the PM should remain intact and protect the facial nerve and brainstem surface. IX = glossopharyngeal nerve, X = vagus nerve, V = trigeminal nerve, SPV = superior petrosal vein. (copyright Arevik Abramyan, used with permission)

**Fig. 2:**
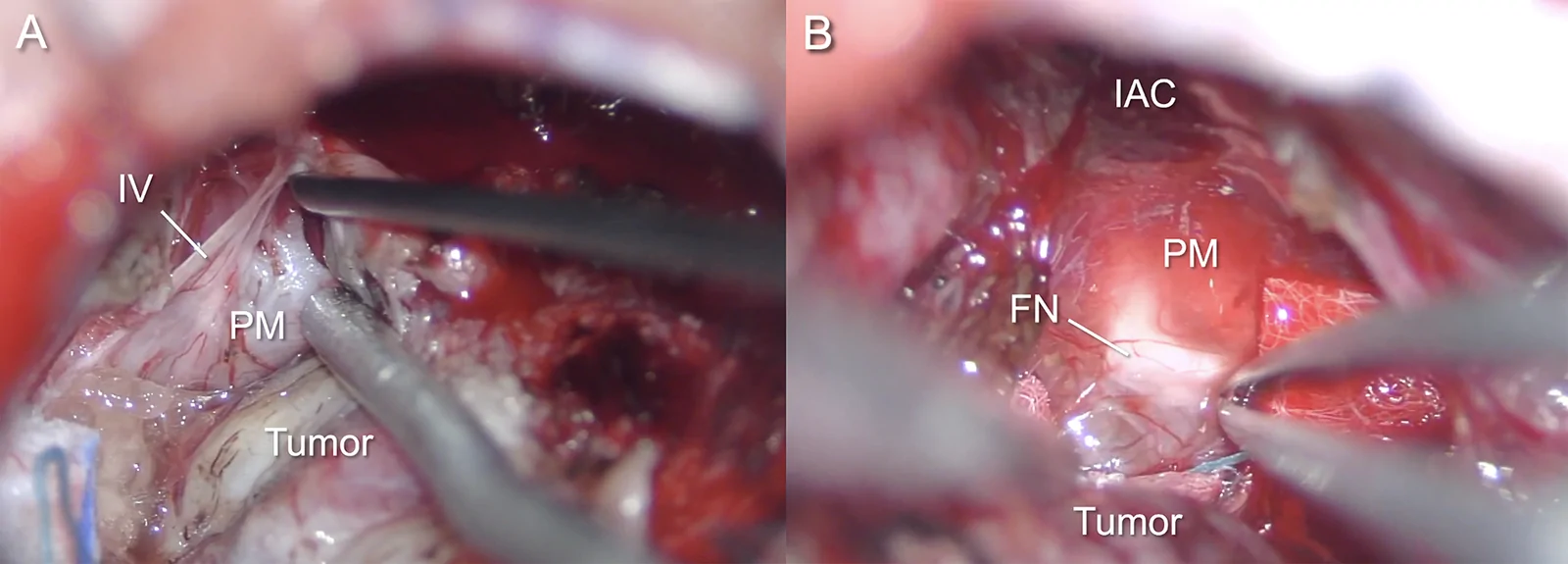
Intraoperative photographs of a left-sided retrosigmoid approach with subperineural dissection. **A**: The inferior pole of the tumor is dissected away from the inferior vestibular nerve (IV) which becomes a thin attenuated perineural membrane (PM). **B**: This membrane appears as a clear semi-transparent membrane (“holy veil”) that provides a protective buffer layer over the facial nerve (FN). As the subperineural plane is developed, the plane of dissection is carried between the PM and the tumor parenchyma. A radical NTR was achieved and the FN stimulated at 0.05 mA. Immediate postoperative facial nerve outcome was HB I. The preoperative and postoperative MRI is seen in Fig. 4**C** and **G**

The subperineural dissection technique (Figs. 1 and 2) performed in this cohort of patients involved identifying the vestibular nerve fibers and dissecting them away from the tumor parenchyma. As the vestibular nerve gets attenuated, this becomes a thin clear membrane called the perineural membrane (PM). The plane of dissection is then carried between the PM and the true tumor parenchyma so that the facial and cochlear nerves are on the opposite side (ventral side) of the PM. The PM serves to act as a protective buffer layer to shield the facial nerve as well as the pial surface of the brainstem. As in the example of onion-peeling, the moist core of the onion represents the true tumor parenchyma, while the outer dry leaves of the onion represent the attenuated vestibular fibers and PM. The subperineural plane of dissection is developed between the moist core of the onion (true tumor parenchyma) and the dry leaves (PM) so that the facial nerve, which resides on the outside of the dry leaves, is protected from direct trauma during dissection. Any tumor adherence to the PM can be released and untethered by sharply trimming the tumor with microscissors and leaving microscopic tumor residue that is adherent to the PM. We propose that this strategy can result in maximal EOR while preserving and optimizing facial nerve function, thus achieving optimal onco-functional balance. If GTR cannot be achieved, then a radical NTR or STR can be accomplished, leaving the least amount of residual tumor while optimizing facial nerve function.

### Extent of resection

The extent of resection was defined in the following manner: GTR (100% removal), NTR (95 to 99% removal), STR (less than 95% removal).

### Data analysis

Statistical analyses were performed using Microsoft Excel (Microsoft, Redmond, WA). Categorical variables were analyzed using the Pearson Chi-Squared or Fisher’s Exact Test. Statistical significance for all analyses was set at *p* < 0.05.

## Results

### Patient demographics and tumor characteristics

A total of 146 consecutive patients in our study underwent surgery for VS using the subperineural technique between September 2009 to February 2025 by a single surgeon (JKL). The patient population had an average age of 54 years (range: 17–82), with 47.3% males (69/146) and 52.7% females (77/146). The mean follow-up time was 89.8 months (range: 15.2 months to 199.7 months). Tumor sizes were categorized using the Koos grading system [12]; 11.0% (16/146) were grade I tumors, 19.2% (28/146) were grade II, 17.8% (26/146) were grade III, and 52.1% (76/146) were grade IV. The mean tumor diameter in its longest axis was 2.24 cm (range: 0.3–6.4 cm, SD: 1.14) and the mean tumor volume measured was 12.9 cm^3^ (range: 0.04–88.32 cm^3^, SD: 18.07).

### Operative approach and extent of resection

**Fig. 3:**
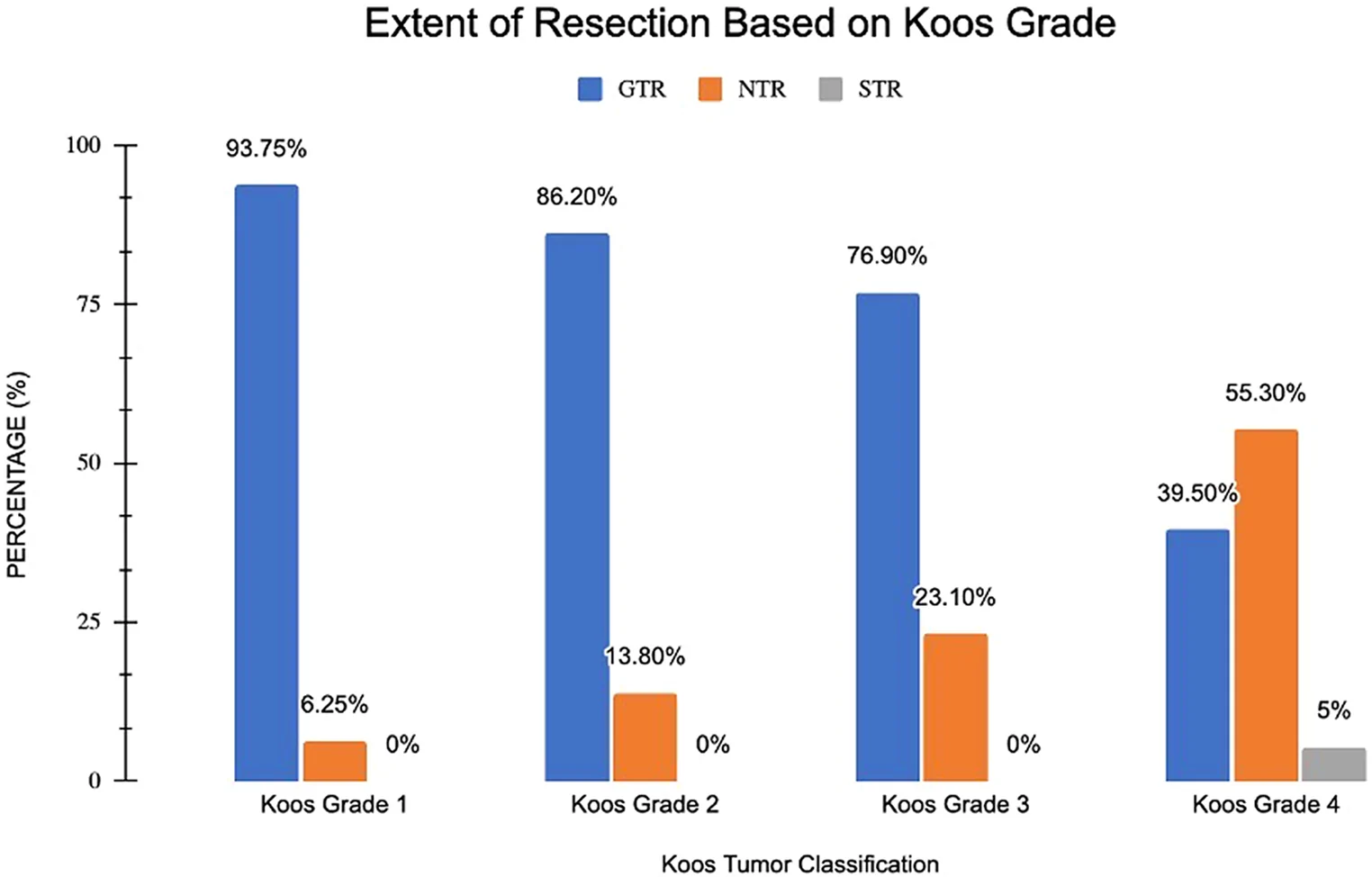
Extent of tumor resection (GTR, NTR, or STR) stratified by Koos Grade. There was a gradual decrease in rate of GTR and increase rate of NTR with increasing Koos Grade and tumor size

The retrosigmoid approach was the most frequently used approach in 86.3% of cases (126/146), followed by the translabyrinthine approach in 13.7% (20/146). GTR was achieved in 61.0% of patients (89/146), NTR in 36.3% (53/146), and STR in 2.7% (4/146) (Fig. 3). GTR decreased and NTR increased with increasing Koos grade I to IV (GTR: 93.75%, 86.2%, 76.9%, 39.5%; NTR: 6.25%, 13.8%, 23.1%, 55.3%, respectively). Continuous intraoperative facial nerve electromyographic monitoring was performed. At the conclusion of tumor resection, the facial nerve at the brainstem was directly stimulated with a Prass probe. The minimum stimulation threshold was recorded and defined as the lowest current (mA) eliciting a recordable response. The overall mean stimulation threshold was 0.062 mA.

### Facial nerve outcomes

Facial nerve function was assessed preoperatively and postoperatively at multiple time points using the HB grading system. Preoperatively, 98.6% of patients (144/146) had HB grade of I and 1.4% (2/146) were HB II. Immediately following surgery, 84.2% (123/146) retained HB grades I-II, whereas 8.9% experienced temporary worsening to immediate post-op HB grades of III-IV (13/146), and 6.8% (10/146) were HB V-VI.

**Fig. 4:**
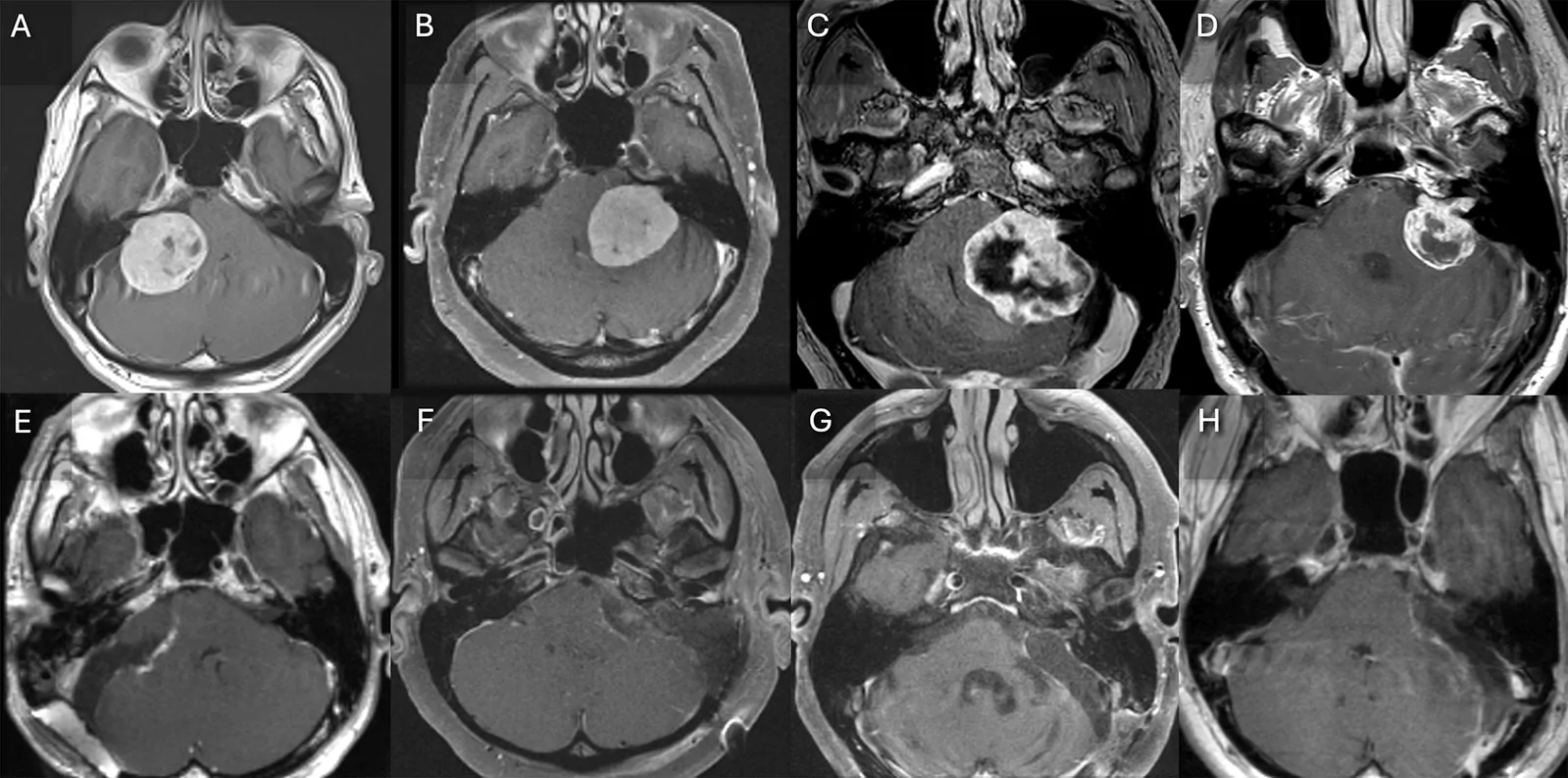
Representative collage of preoperative (**A**-**D**) and respective postoperative (**E**-**H**) MRI scans demonstrating patients with VS who underwent GTR and NTR with HB I-II outcomes. All patients shown in this figure had NTR except the patient in **D** and **H** who had GTR. Many patients who had NTR did not have radiographically visible nodular tumor (**E**-**G**)

At the most recent follow-up, 95.2% of patients (139/146) had recovered to HB grades I-II, while only 4.8% (7/146) had persistent facial nerve dysfunction (HB grades III-VI). When stratified by Koos grade, favorable HB I-II outcomes were achieved in 100% of Koos I-II and 93.1% of Koos III-IV tumors (Table 1). There was no significant impact of EOR (GTR vs. NTR) on HB I-II facial nerve outcomes (97.8% vs. 92.5%, *p* = 0.196). Figure 4 demonstrates a wide variety of representative Koos grade IV tumors who underwent NTR or GTR with a facial nerve outcome of HB I-II. Many patients who underwent NTR did not have visible nodular enhancing tumor on the postoperative MRI (Fig. 4E-G).

**Table 1:** Facial nerve outcomes based on koos grade tumor size

Tumor Grade	HB I-II [*n*](%)	HB III-IV [*n*](%)	HB V-VI [*n*](%)	Total [*n*](%)
Koos Grade I	16 (100.0)	0 (0.0)	0 (0.0)	16 (11.0)
Koos Grade II	28 (100.0)	0 (0.0)	0 (0.0)	28 (19.1)
Koos Grade III	25 (96.1)	1 (3.9)	0 (0.0)	26 (17.8)
Koos Grade IV	70 (92.1)	5 (6.6)	1 (1.3)	76 (52.1)
Total	139 (95.2)	6 (4.1)	1 (0.7)	146 (100.0)

### Interval time of facial nerve recovery

From the 8.9% (13/146) cohort of our patients who had immediate postoperative HB III-IV, 92.3% recovered to HB grade of I-II (12/13) within an average time of 6.3 weeks (range 5.3–12 wks). One patient who was immediate postoperative HB IV improved to HB III at 12 weeks postoperative follow-up. From the 6.8% (10/146) of patients who had immediate postoperative HB grade of V-VI, 40% recovered to HB grade I-II (4/10) within an average of 13.2 weeks (range 6–24 weeks), 50% improved to HB grade III-IV (5/10) within an average of 9.1 weeks (range 6–48 weeks), and 10% remained at HB grade VI (1/10). None of the patients who were in the immediate postoperative HB I-II group worsened to a higher category (III-VI) at the most recent follow-up visit.

Facial nerve stimulation thresholds were documented intraoperatively at the conclusion of tumor resection and yielded a mean stimulation threshold of 0.062 mA overall. Patients achieving HB grades I–II had a mean stimulation threshold of 0.059 mA (SD ± 0.023; range 0.05–0.2). In contrast, patients with HB grades III–VI (*n* = 7) required significantly higher stimulation levels, averaging 0.129 mA (SD ± 0.076; range 0.05–0.25) (*p* = 0.0003, Mann-Whitney test).

### Tumor recurrence

The overall tumor recurrence in this study was 3.4% of patients (5/146). Tumor recurrence occurred in 1.1% of GTR cases (1/89) and 7.5% of NTR cases (4/53). Although recurrence was lower after GTR, this difference did not reach statistical significance (Fisher’s exact *p* = 0.0646). Three of these patients received SRS. Although 2 of these remained stable after SRS, one patient continued to have tumor growth and progression after SRS and required re-operation. One patient refused SRS and another patient relocated to a different country and was lost to follow-up. Two additional patients received SRS to the residual tumor that did not have tumor recurrence nor progression and remained stable post-SRS.

### Postoperative complications

Postoperative complications were observed in 9.6% of patients (14/146). CSF leak (incisional or rhinorrhea) was observed in 2.7% of patients (4/146). Graft site complications included 2 patients with fat graft hematoma (1.4%) and 3 patients with graft site incision breakdown (2.1%). Cerebellar edema was observed in 2 patients (1.4%) attributed to excessive retraction during the retrosigmoid approach. Cranial incisional wound breakdown was observed in 1 patient (0.7%). Transient CN VI nerve palsy was observed in 1 patient (0.7%). There was one mortality (0.7%) from intracranial hemorrhage following anticoagulation for postoperative myocardial infarction.

## Discussion

This study demonstrates that optimal onco-functional balance with favorable facial nerve outcomes and maximal safe resection of VS can be achieved using the subperineural “onion-peeling” dissection technique. *In our series*,* 95.2% of patients achieved HB I-II scores while obtaining an EOR ≥ 95% (GTR/NTR) in 97.3% of patients.* The rate of tumor recurrence remained low at 3.4%. The key technical nuance to achieving better facial nerve function is identifying the PM of the vestibular nerve and maintaining the plane of dissection between the PM and the true tumor parenchyma. This allows the PM to serve as a protective barrier between the facial nerve and the surgical dissecting instruments, theoretically avoiding direct contact and potential trauma from mechanical and vascular injury to the facial and cochlear nerves. This concept was introduced by Sasaki et al. [14], whose histological analysis of VSs identified a thin connective tissue layer on the tumor surface comprised of attenuated vestibular nerve fibers and the perineurium. Thus, the ideal cleavage plane for surgical dissection is between the perineurium (or attenuated vestibular nerve fibers) and the tumor parenchyma. Because the facial and cochlear nerves reside on the ventral side of this perineurium (which we prefer to call the PM), dissection along the vestibular nerve-tumor interface via subperineural dissection can potentially optimize facial and cochlear functions. Moreover, maintaining a protective layer of the PM can reduce mechanical and vascular injury to the cranial nerves and maximize safe tumor resection. This concept has been further demonstrated in several single case operative video publications [14–18]. To our knowledge, this current study represents the first report on optimizing onco-functional outcomes by maximizing the EOR in a series of VS patients while optimizing facial nerve function using the subperineural dissection technique with the intent of GTR.

The PM can be identified at both superior and inferior poles of the tumor by identifying the vestibular nerve tissue. Careful peeling of the vestibular nerve from the tumor capsule can eventually reveal a thin clear membrane that is reminiscent of arachnoid. Despite its resemblance to arachnoid tissue, histological evaluation reveals that this layer is, in fact, the PM. This membrane is what we call the “holy veil” since it is a semi-transparent membrane where one can sometimes see the facial nerve protected behind it (Fig. 2). Microdissection is carried along the tumor capsule and PM. Any tumor tissue that is strictly adherent to the PM is sharply trimmed with microscissors, leaving only minimal tumor residue to achieve a maximal safe resection.

### Technical nuances of subperineural dissection and intraoperative decision for NTR

In VS surgery, the facial nerve can be displaced anteriorly, anterosuperiorly, anteroinferiorly, superiorly, or inferiorly, and its course may vary substantially depending on the tumor configuration and extension. In our experience, we have noted that the superior and anterosuperior displacement pattern of the facial nerve tends to place the facial nerve more vulnerable to traction and dissection injury, and subsequent postoperative palsy. As such, we have a lower threshold to sharply trim the tumor, leaving it adherent to the PM overlying the course of the facial nerve, instead of attempting a GTR. If needed, a subcapsular dissecting plane can be created, leaving more tumor tissue on areas of PM adherence in order to preserve FN function.

We prefer to initially control the lateral pole of the tumor in the IAC and peel the tumor off the PM within the IAC. Here the nerves are more robust and not under as much stretch as the nerve in the cisternal segment. Care should be taken if the facial nerve is displaced superiorly along the wall of the IAC. We typically use microscissor dissection and a disc dissector to peel the tumor away from the PM. If there is any tumor adherence or transient EMG irritation (A-trains), we trim the tumor, leaving a thin rim attached to the PM and continue the dissection in a subcapsular (subparenchymal) fashion. If an A-train is encountered during dissection, dissection is halted immediately and the field is irrigated with warm Lactated Ringer’s solution. If the train resolves, and facial MEP is stable, we continue the dissection with a low threshold to convert to a subcapsular plane of dissection, leaving a thin residue on the PM. The PM can be picked up in other areas of the dissection (medial pole, superior pole, or lateral pole). It is important to avoid any sustained A-train lasting longer than 10 seconds as this can potentially result in postoperative facial nerve palsy. We have found that the zone between the porus acusticus and the distal cisternal segment in the cerebellopontine angle is where the tumor tends to be most adherent to the PM. This is an area where we frequently leave a thin tumor residue to achieve an NTR. After intratumoral debulking, we routinely find the PM at the inferior pole of the tumor where the inferior vestibular nerve fibers are identified and peeled away from the tumor parenchyma to develop the PM. We feel that this is the safest area to start as opposed to the superior pole, where the facial nerve can be more vulnerable to injury.

### Critical analysis of the adaptive hybrid approach (planned STR and SRS)

The adaptive hybrid approach, characterized by pre-planned intracapsular STR followed by SRS, has gained popularity due to concerns about facial nerve injury. In a recent meta-analysis of 248 patients by Starnoni et al.,^19^ 96.1% of patients achieved HB I-II outcomes with an overall tumor control rate of 93.9% with a mean follow-up of 46 months (range: 28-68.8 months). Although the initial results appear favorable, there are several concepts that should be addressed. First, the resection strategy is primarily intracapsular debulking, with no attempt at extracapsular dissection and removal [2, 11]. By exclusively focusing on intracapsular tumor removal, one can underestimate the amount of residual tumor, even with image-guided intraoperative navigation, thereby leading to a wide range of STR volumes from 1% to 95% of the original tumor volume. This can result in less favorable residual tumor volumes and potentially higher tumor recurrence rates. The inability to precisely define a consistent operative endpoint increases the likelihood of underestimating postoperative residual tumor volume and overestimating the EOR, leading to less optimal results.

Several studies have shown that tumor recurrence rates correlate directly with the volume of residual tumor, suggesting that EOR plays a significant role in long-term tumor control [8]. A study by Bloch et al. [20] focusing on recurrence rate after STR and NTR showed that STR resulted in 12 times greater risk of tumor recurrence when compared to NTR. Another study by Monfared et al. [7] focusing on large VS and degree of EOR have found that tumor regrowth is three times more likely to occur when patients undergo STR when compared to NTR or GTR. Volumetric analysis has demonstrated that STR results in a 13-fold increased risk of tumor recurrence compared to NTR, while residual nodular enhancements following STR increase the likelihood of recurrence by 16-fold compared to GTR/NTR [21, 22].

Additionally, the hybrid approach has diverted attention from maintaining a high level of advanced microsurgical cranial nerve dissection techniques. By prioritizing STR that is primarily intracapsular, there is no attempt at trying to clear the tumor from the PM interface. If the teachers and mentors of training centers are not performing advanced cranial nerve microdissection, how will the future generation of trainees learn and maintain a high level of proficiency and expertise? The craft of microneurosurgery, particularly methods like subperineural dissection for VS surgery, is in jeopardy of becoming a lost art. As more surgeons increasingly opt for the hybrid STR-SRS approach, the ability to perform meticulous, nerve-preserving tumor resection of other complex tumors in the CPA and petroclival region is likely to see a sharp decline. This trend may lead to a future where neurosurgeons lack the confidence and experience to perform a maximal GTR/NTR, thereby perpetuating a reliance on hybrid STR/SRS techniques. If this trend persists, the field risks diminishing the overall quality of care for VS patients, with fewer surgeons feeling equipped to take on complex cases to achieve optimal onco-functional outcomes.

Lastly, the long-term follow-up from the hybrid approach is also lacking (mean follow-up of 46 months). Therefore, we do not know how these tumors will behave in the next 10 to 20 years. Currently the recurrence rate from this approach is 7.6% and the risk of recurrence after longer-term follow-up remains unknown [9, 11, 23, 24]. If there is future tumor recurrence after SRS that may need further re-operation, the surgical risk for facial nerve damage is likely to increase based on the patient having both prior surgery and radiation. With the large number of hybrid treated tumors, we could be creating a “new disease entity” in the next several decades to come, complicating future treatment with potentially increased morbidity. It has been shown that the surgery of VS after failed SRS has a higher risk of facial nerve morbidity [25]. One can postulate that the risk would be even higher for surgery on VS that have undergone both surgery and SRS. In our study, we had one patient who had continued tumor progression after surgical resection and SRS to a growing residual tumor remnant. She underwent repeat surgical resection using the subperineural dissection technique. Immediate postoperatively, she had very minimal facial palsy HB II which improved to HB I within less than 6 weeks after surgery. This suggests that subperineural dissection can be performed safely despite prior surgery with SRS for failed recurrences.

### Comparative analysis of planned STR-SRS hybrid approach versus intended maximal safe resection using the subperineural dissection technique

While the hybrid approach promotes a “nerve-centered” strategy, our study demonstrates that comparable excellent facial nerve outcomes while maintaining maximal safe resection can be achieved in large Koos Grade III/IV tumors using the subperineural “onion-peeling” dissection technique (Table 2). Compared to the hybrid approach, our method maintains a high rate of excellent facial nerve outcomes (93.1% HB I-II), achieves a high extent of resection (GTR/NTR rates of 96.1%), significantly reduces reliance on adjuvant SRS (4.9% vs. 100%), and has a slightly lower tumor recurrence rate (4.9% vs. 7.6%). Unlike the hybrid method, where the STR is pre-planned and resection is purely intracapsular, our approach of intended maximal safe resection has the philosophy of pre-planned intended GTR using extracapsular subperineural dissection. The decision to leave residual tumor behind is based on intraoperative judgement (“gametime decision”), and if so, our goal is to leave the least amount of residual tumor volume adherent to the PM so that in essence, we achieve a radical NTR. Therefore, we postulate that the best attempt at GTR/NTR can also be a facial “nerve-sparing” or “nerve-centered” strategy. Further supporting the efficacy of the subperineural approach, Wu et al. conducted a retrospective study on 124 patients undergoing VS resection via the subperineural dissection technique [26]. Their findings align with our results, demonstrating an 88.7% of HB I-II outcomes and a GTR rate of 83.9%.

**Table 2:** Subgroup analysis of Koos III/IV tumors of current series (intended GTR, maximal safe EOR) vs. meta-analysis of hybrid planned STR + SRS (Starnoni et al.) [19] for large VS

	Current Series, Koos III/IV (Intended GTR/NTR, Maximal Safe EOR)	Meta-Analysis of Hybrid Planned STR + SRS (> 3 cm, Koos IV) (Starnoni et al.) [19]
HB 1–2 FN outcome	93.1%	96.1%
GTR/NTR	96.1%	0%
STR	3.9%	100%
Recurrence	4.9%	7.6%
SRS Usage	4.9%	100%
Mean Follow-up (months)	87.8 (15.2-199.7)	46 (22-68.8)

Table 2 compares the clinical and functional outcomes reported by Starnoni et al. [19] with those of our current study. Among our Koos grade III and IV patients, we were able to achieve a high rate of good facial nerve outcomes (HB I-II: 93.1%), comparable to the results reported by Starnoni et al. (HB I-II: 96.1%).

The subperineural technique can also be applied not only to the facial nerve but also to the meticulous preservation of the cochlear nerve and its vascular supply for the goals of hearing preservation in patients with smaller tumors and preoperative serviceable hearing status. Khanna et al. described their technique of preserving vestibular nerve fibers during resection of a VS resection that resulted in hearing preservation (word recognition score of 84% preoperatievely to 58% postopratively) [27]. In a study of 10 VS patients with smaller tumors measuring 10–20 mm, Labib et al. also reinforced this concept, demonstrating that intentional preservation of the vestibular nerve led to 100% facial nerve preservation (HB I) and 70% hearing preservation [28]. This supports the concept of preserving the PM to optimize hearing preservation and facial nerve function.

Our study reveals several key advantages of the subperineural technique. Our findings indicate that patients who undergo subperineural dissection experience a high rate of immediate postoperative HB I-II outcomes, and if there is moderate postoperative facial weakness, there is a quicker return to HB I-II levels, especially during the first 6 weeks after surgery. Schneider et al. found that NTR (10/10 patients HB grade I-II) provided a better facial nerve preservation compared to GTR (21/27 patients HB grade I-II), without significantly increasing recurrence risk, but their study was limited by a smaller sample size and a mean follow up time of only 36 months [29]. We found no significant differences in HB I-II outcomes between GTR (97.7%, 87/89) and NTR (92.4%, 49/53), which supports the idea that facial nerve preservation outcomes can be similar whether the EOR is GTR or NTR.

### Challenges and future considerations for skull base surgeons

Nevertheless, the subperineural dissection technique is associated with its own set of limitations. It requires surgical understanding of the tumor-nerve interface, meticulous intraoperative judgement, and flexibility in surgical objectives based on real-time observations. This approach is inherently more technically demanding compared to the pre-planned STR approach and therefore requires substantial microsurgical expertise. Thus, it is important for training programs to continue to raise the bar for VS surgery and development of advanced microsurgical skills. The aptitude to proficiently execute GTR or NTR with both safety and efficacy must remain a fundamental goal for every skull base surgeon, and this is only achievable through continuous practice, training and mentorship. It is interesting to note that the rate of GTR decreased and rate of NTR increased with increasing tumor size (Fig. 3), which reflects the senior author’s lower threshold to leave microscopic residual tumor behind to obtain better facial nerve outcomes in larger complex tumors.

### Limitations

This study reflects outcomes from a single-surgeon from a high-volume skull base center and may not be generalizable to broader populations with varying microsurgical expertise. The subperineural “onion-peeling” dissection technique is technically demanding and has a steeper learning curve. The decision to leave residual tumor behind relies on intraoperative judgment, which may lead to variability in outcomes based on surgical experience. The study is also limited in that it is a retrospective case series, necessitating larger, multi-center studies with longer-term follow-up to further validate the technique’s efficacy. Follow-up durations were variable, which may impact the accuracy of long-term recurrence rates. Despite achieving high rates of GTR and NTR, recurrence risk remains, emphasizing the need for longer follow-up studies.

While hearing preservation is an important goal in contemporary VS surgery in patients with serviceable preoperative hearing (AAO-HNS Class A or B), this study focused specifically on facial nerve outcomes and extent of resection. The vast majority of patients in this study were larger Koos grade III and IV tumors with non-serviceable hearing (Class C or D) preoperatively, making facial nerve preservation the primary functional goal. Nevertheless, we acknowledge that a comprehensive analysis of hearing preservation in appropriate cases is essential, and this data is currently being collected for future analysis. Future prospective, multi-institutional studies with standardized follow-up are necessary to establish the long-term benefits and reproducibility of the subperineural dissection approach.

## Conclusion

Maximal safe tumor resection with minimal neurological morbidity remains the cornerstone of VS surgical management. This study demonstrates that the subperineural “onion-peeling” dissection technique provides a precise and effective method for maximizing tumor removal while optimizing facial nerve function by utilizing the PM as a protective buffer for facial nerve protection, thereby achieving an *excellent onco-functional outcome*. Importantly, the decision to leave residual tumor should be guided by intraoperative observation and nuanced clinical judgement. Our “nerve-sparing centered” approach is a safe and effective strategy for VS surgery underscoring the need to continuously advance the benchmarks of advanced microsurgical techniques.

## Data Availability

No datasets were generated or analysed during the current study.
